# Combined influence of poor health behaviours on the prevalence and 15-year incidence of age-related macular degeneration

**DOI:** 10.1038/s41598-017-04697-3

**Published:** 2017-06-28

**Authors:** Bamini Gopinath, Gerald Liew, Victoria M. Flood, Nichole Joachim, George Burlutsky, Paul Mitchell

**Affiliations:** 1 Centre for Vision Research, Westmead Institute for Medical Research, University of Sydney, Sydney, NSW Australia; 2 Faculty of Health Sciences, University of Sydney, Sydney, NSW Australia; 3 Westmead Hospital, Western Sydney Local Health District, Westmead, NSW Australia

## Abstract

We aimed to establish the collective influence of four lifestyle practices (physical activity, diet, smoking and alcohol consumption) on the prevalence and incidence of AMD. At baseline, 2428 participants aged 49+ with complete lifestyle and AMD data were examined, and of these, 1903 participants were re-examined 15 years later. AMD was assessed from retinal photographs. A health behaviour score was calculated, allocating 1 point for each poor behaviour: current smoking; fruits and vegetables consumed <4 serves daily; <3 episodes of physical activity per week; and >2 alcoholic drinks per day. Cross-sectional analysis showed that participants who engaged in all 4 poor health behaviours (n = 29) versus those who did not engage in unhealthy behaviours (reference group; n = 677) had greater odds of any and late AMD: multivariable-adjusted OR, 5.14 (95% CI, 1.04–25.45) and OR 29.53 (95% CI 2.72–321.16), respectively. A marginally non-significant association was observed between increasing number of poor health behaviours and 15-year incidence of early AMD (multivariable-adjusted *P*-trend = 0.08). Our data suggests that motivating patients with AMD to eat better, exercise more, limit alcohol intake and avoid smoking seems advisable to decelerate the development or worsening of existing AMD.

## Introduction

Age-related macular degeneration (AMD) is a progressive, chronic disease of the central retina, and is a leading cause of blindness and low vision among older adults^1^. Anti-angiogenic therapies have proven to be effective in reducing visual loss in those with neovascular AMD; and the use of high-dose antioxidant supplements have been shown to slow the progression of intermediate to advanced disease^2^. The results of the Age-Related Eye Disease Studies (AREDS) demonstrated that the disease process can be impacted by nutritional interventions^2–4^. It is important to note that AREDS supplements are useful in slowing the progression of existing disease but do not appear to prevent development of AMD^5^. Moreover, the benefits or safety of using such high-dose antioxidants for long periods, as might be needed in patients with AMD, has not been established^2, 6^.

Therefore, preventive measures through lifestyle modifications are attractive strategies, because these are more affordable than clinical therapies, and do not require specialists for administration^7^. By reducing or eliminating modifiable risk factors, the likelihood that AMD will develop or progress (particularly to its late stage) could be reduced or its age at onset delayed. Specifically, a healthy diet^2, 5, 8–10^, not smoking^11, 12^, abstaining from alcohol^13^, and physical activity^9, 14^ have been previously associated with lower occurrence of early or advanced AMD, or both, in epidemiologic studies^2^.

The magnitude of risk reduction associated with multiple healthy or unhealthy lifestyles, considered jointly, may be greater than the magnitude associated with individual healthy/unhealthy lifestyle factors^2^. However, to our best knowledge, only the Carotenoids in Age-Related Eye Disease Study (CAREDS) has examined the association between a combination of three healthy behaviours (healthy diet, physical activity, and not smoking) and risk of AMD in women^2, 9^. CAREDS found that women who had a combination of these three healthy lifestyle factors had a 3-fold lower odds for early AMD relative to women who had unhealthy lifestyles^9^. A more recent report from CAREDS showed that having unhealthy lifestyles and two complement factor H risk alleles (genetic risk factors for AMD) increased AMD risk, in an additive or synergistic manner. However, unhealthy lifestyles increased AMD risk regardless of AMD risk genotype^2^.

It is important to note that the CAREDS cohort comprised of women only and the AMD outcome was a prevalence estimate in this study^2, 9^. Therefore, further research is needed to advance our understanding of how a combination of lifestyle practices, rather than focusing on a single health behaviour, might prospectively influence AMD risk. Hence, we examined the collective influence of smoking, diet, alcohol intake, and physical activity on the prevalence and 15-year incidence of AMD in men and women from a population-based study with participants aged 49 years and over.

## Methods

### Study population

The Blue Mountains Eye Study (BMES) is a population-based cohort study of common eye diseases and other health outcomes in a suburban Australian population located west of Sydney. Study methods and procedures have been described elsewhere^15^. Baseline examinations of 3654 residents aged >49 years were conducted during 1992-4 (BMES-1; 82.4% participation rate). Surviving baseline participants were invited to attend examinations after 5- (1997-9, BMES-2), 10- (2002-4, BMES-3), and 15 years (2007-9, BMES-4) at which 2334 (75.1% of survivors), 1952 participants (75.6% of survivors) and 1149 (55.4% of survivors) were re-examined, respectively. For the current report we have analyzed data from BMES-1 through to -4. The University of Sydney and the Western Sydney Area Human Ethics Committees approved the study including all methods that were performed, and written informed consent was obtained from all participants at each examination. All methods in this study were performed in accordance with the relevant guidelines and regulations.

### Assessment of AMD

We took two 30° stereoscopic color retinal photographs of the macula of both eyes, which were graded for presence of early and late AMD using the Wisconsin AMD Grading System^16, 17^. Inter- and intra-grader reliability showed good agreement for grading of specific AMD lesions with quadratic weighted kappa values ranging from 0.64 to 0.93 and 0.54–0.94 respectively^18^. The detailed methodology of AMD ascertainment in this population has been previously reported^16, 17^. Early AMD was defined as the absence of late AMD and presence of either: 1) large (>125-µm diameter) indistinct soft or reticular drusen or 2) both large distinct soft drusen and retinal pigmentary abnormalities (hyperpigmentation or hypopigmentation) in either eye^17^. Similarly, late AMD was defined as the presence of neovascular AMD or geographic atrophy in either eye^17^. Any AMD was defined as having early or late AMD. A retinal specialist (P.M.) adjudicated all uncertain retinal pathology and confirmed all late AMD cases.

### Assessment of health behaviours

An interviewer-administered questionnaire was used to collect information on a wide range of health behaviours. We used similar methods to define health behaviours and to generate an index ranging from 0 to 4, as detailed in a previous report from the BMES^19^. Briefly, smoking behaviour was divided into two categories: current and former/never smoker. Poor smoking behaviour was defined as being a current smoker, that is, those who reported that they currently smoked or had stopped smoking <1 year before the examination. Alcohol intake was assessed by questions about the frequency of consuming alcoholic drinks (days/week), the usual number of drinks on a day when alcohol was consumed, and the usual type of alcohol (beer, wine, port, or spirits). People were divided into two categories (≤2 drinks/day and >2 drinks/day). These categories were formulated based on the recent recommendations of the Australian National Health and Medical Research Council^20^ of up to 2 standard drinks a day. Poor drinking behaviour was defined as >2 drinks/day. The amount of alcohol per drink is the same, that is, a standard serve of alcohol = 10 g. Participants provided details of walking exercise and the performance of moderate or vigorous activities^21^, Participants were asked how many times in the last 2 weeks and the estimated time (in hours and minutes) that they spent walking, doing vigorous activity and/or leisure time activities. We defined poor physical activity as <3 episodes/week.

Dietary data were collected using a 145-item self-administered food frequency questionnaire (FFQ). The FFQ is modified for Australian diet and vernacular from an early Willett FFQ^22^ and including reference portion sizes. Participants used a 9-category frequency scale to indicate the usual frequency of consuming individual food items during the past year. Foods listed in the FFQ were categorized into major food categories and subcategories similar to those used for the 1995 Australian National Nutrition Survey^23^. The cut-points used for analyses of fruit and vegetable consumption were based on the overall recommended daily intake (RDI) for each food group^24^. One serve of vegetables and fruits is specified as 75 grams and 150 grams, respectively^24^. Poor dietary behaviour was defined as having had fruits and/or vegetables less than 4 serves daily.

A health behaviour score was calculated based on the four poor health behaviours: cigarette smoking, high alcohol intake, physical inactivity, and a low fruit and vegetable intake. Participants scored 1 point for the presence of each of the poor health behaviours. The poor health behaviour score ranged from 0 (no poor health behaviours) to four (all four poor health behaviours).

### Assessment of covariates

For the current study, only baseline covariate information was used in the analyses, i.e. covariate information collected during BMES-1 (1992-4). The covariates that were included in the final, multivariable model were those that were previously identified as risk factors for AMD in the BMES cohort (in addition to age and sex), including: white cell count^25^, and fish consumption^26^. Fasting blood samples collected at BMES-1 were also processed for white cell count. We extracted separate data on the frequency of consuming fish, including salmon, tuna and sardines from the food frequency questionnaire^27^.

### Statistical analyses

SAS statistical software (SAS Institute, Cary NC) version 9.3 was used for analyses including t-tests, ${\chi }^{2}$ -tests and logistic regression. Associations between individual and collective number of poor health behaviours (smoking, diet, alcohol intake and physical activity) and prevalence of AMD (study outcome) were examined in logistic regression models, adjusted for age, sex, white cell count, and fish consumption; and expressed as odds ratios (OR) and 95% confidence intervals (CI). Associations between individual and collective number of poor health behaviours and 15-year incidence of AMD (study outcome) were examined in discrete linear logistic regression models, adjusted for age, sex, white cell count, and fish consumption; and expressed as hazard ratios (HR) and 95% CI.

## Results

Of the 3654 participants examined at baseline, 2428 participants had complete information on AMD and lifestyle behaviours and thus, were included in prevalence analysis. Of these, 1903 participants with complete AMD and lifestyle data were examined 15 years later. Table 1 shows the baseline characteristics of the whole cohort as well as study participants stratified by number of poor health behaviours. Those participants who engaged in all 4 poor lifestyle choices were more likely to be male, younger, smoked, have higher white cell count, poor physical activity and higher alcohol consumption but lower intakes of fruits, vegetables and fish. Initially, we assessed the associations between each individual unhealthy behaviour and prevalence and incidence of AMD (Table 2). Current smoking was associated with 1.98- and 5.98-fold increased odds of prevalent early and late AMD, respectively. Current smoking at baseline increased the risk of incident late AMD but not early AMD 15-years later. Higher levels of alcohol intake was significantly associated with both the prevalence and incidence of early AMD: OR 1.68 (95% CI 1.00–2.82) and OR 1.39 (95% CI 1.01–1.91), respectively. Having had fruits and/or vegetables less than 4 serves daily was associated with the prevalence of late AMD only, OR 3.87 (95% CI 1.80–8.32). Physical activity was not associated with either prevalence or incidence of AMD (Table 2).

**Table 1:** Baseline characteristics of study participants stratified by number of poor health behaviours in the Blue Mountains Eye Study (n = 2428).

Characteristics	All	No. of poor health behaviours, n (%)					
		0 (n = 677)	1 (n = 1082)	2 (n = 503)	3 (n = 137)	4 (n = 29)	P-value^1^
Age, *yrs*	65.3 (9.3)	65.6 (9.1)	66.3 (9.1)	63.8 (8.8)	63.1 (8.7)	60.1 (6.1)	<0.0001
Male sex	1191 (49.1)	302 (40.8)	503 (41.7)	277 (50.5)	92 (61.3)	17 (58.6)	<0.0001
Current smoking	378 (15.6)	0 (0.0)	69 (5.7)	177 (32.2)	103 (68.7)	29 (100.0)	<0.0001
Poor physical activity	1492 (61.4)	0 (0.0)	887 (73.6)	444 (80.9)	132 (88.0)	29 (100.0)	<0.0001
White cell count, x *10* ^*9*^ */L*	6.5 (1.72)	6.4 (1.6)	6.6 (1.7)	7.0 (1.9)	7.5 (1.9)	7.5 (1.9)	<0.0001
Fruits, *g*/*day*	342.6 (255)	430.9 (305)	355.4 (239)	246.4 (210)	134.0 (140)	63.9 (49)	<0.0001
Vegetables, *g*/*day*	442.8 (194)	482.8 (182)	460.3 (190)	400.5 (193)	357.9 (148)	330.9 (100)	<0.0001
Alcohol, *g*/*day*	10.6 (16.4)	4.1 (5.2)	7.6 (12.7)	17.9 (20.6)	33.2 (22.9)	43.8 (16.6)	<0.0001
Fish consumption (≥1 serve/week)	1591 (59.5)	492 (66.4)	728 (60.4)	286 (52.1)	73 (48.7)	12 (41.4)	<0.0001
15-year incidence of AMD							
Early	248 (15.2)	72 (14.6)	111 (15.2)	53 (16.8)	9 (12.3)	3 (15.8)	0.87
Late	76 (4.0)	18 (3.2)	35 (4.1)	17 (4.6)	5 (5.6)	1 (5.0)	0.75

Data are presented as means ± SD or n (%). *Unadjusted p values from test of heterogeneity across categories of health behaviours.

**Table 2:** Individual health behaviours in relation to the prevalence and 15-year incidence of AMD in the Blue Mountains Eye Study.

Health behaviour	Prevalence, odds ratio (95% CI)		Incidence, hazard ratio (95% CI)	
	Early AMD (n = 106)	Late AMD (n = 42)	Early AMD (n = 248)	Late AMD (n = 76)
Current smoking vs never and former smoking	1.98 (1.07–3.67)	5.98 (2.44–14.65)	1.31 (0.84–2.02)	2.29 (1.19–4.40)
Alcohol intake, >2 drinks/day vs ≤2 drinks/day	1.68 (1.00–2.82)	1.52 (0.61–3.76)	1.39 (1.01–1.91)	0.93 (0.49–1.74)
Physical activity, <3 episodes/week vs ≥3 episodes/week	1.29 (0.84–2.00)	1.00 (0.49–2.05)	0.96 (0.75–1.24)	1.01 (0.64–1.61)
Fruit and vegetable intake, <4 serves/day vs ≥4 serves/day	1.16 (0.67–2.01)	3.87 (1.80–8.32)	1.21 (0.87–1.70)	1.57 (0.91–2.72)

CI – confidence interval; ^a^Adjusted for age, sex, white cell count, and fish consumption. ^b^One serve of vegetables and fruits is specified as 75 grams and 150 grams, respectively^24^. Poor dietary behaviour was defined as having had fruits and/or vegetables less than 4 serves daily.

Table 3 shows the association between the collective influence of all 4 poor health behaviours and prevalence of AMD. In comparison with people with no poor health behaviours, the odds of any, early and late AMD rose as the number of poor health behaviours increased. There was some variation in the strength of these gradients, although all tests for trend were statistically significant. Thus, in the fully adjusted model, the OR (95% CI) for 4 poor health behaviours compared with none for any AMD was 5.14 (95% CI 1.04–25.45), whereas the corresponding effect estimates for early and late AMD were 2.81 (95% CI 0.34–23.09) and 29.53 (95% CI 2.72–321.16), respectively (Table 3).

**Table 3:** Collective health behaviours and prevalence of AMD in the Blue Mountains Eye Study (n = 2428).

No. of poor health behaviours	Adjusted odds ratio (95% confidence interval)^a^		
	Any AMD (n = 148)	Early AMD (n = 106)	Late AMD (n = 42)
0 (n = 677)	1.0 (reference)	1.0 (reference)	1.0 (reference)
*n (%*)	*30* (*4.4*)	*25* (*3.7*)	*5* (*0.7*)
1 (n = 1082)	1.16 (0.72–1.89)	1.03 (0.60–1.75)	1.86 (0.66–5.27)
*n* (*%*)	*67* (*6.2*)	*45* (*4.3*)	*22* (*2.0*)
2 (n = 503)	1.84 (1.05–3.22)	1.53 (0.82–2.83)	3.23 (1.01–10.32)
*n* (*%*)	*33* (*6.6*)	*23* (*4.7*)	*10* (*2.0*)
3 (n = 137)	4.02 (1.88–8.61)	3.14 (1.34–7.34)	7.93 (1.87–33.58)
*n* (*%*)	*16* (*11.7*)	*12* (*9.0*)	*4* (*5.6*)
4 (n = 29)	5.14 (1.04–25.45)	2.81 (0.34–23.09)	29.53 (2.72–321.16)
*n* (*%*)	*2* (*6.9*)	*1* (*3.6*)	*1* (*3.5*)
*P-value* for linear trend	0.0001	0.01	0.001

^a^Adjusted for age, sex, white cell count, and fish consumption.

Table 4 shows the association between the combined influence of poor health behaviours and the 15-year incidence of AMD. A marginally non-significant association was observed between an increase in the number of poor health behaviours and increased risk of incident early AMD (*P*-trend = 0.08), after adjusting for all potential confounders. Non-significant associations were observed with 15-year incidence of late AMD.

**Table 4:** Collective health behaviours and the 15-year incidence of AMD in the Blue Mountains Eye Study during 1992-4 to 2007-9 (n = 1903).

No. of poor health behaviours	Adjusted hazard ratio (95% confidence interval)^a^	
	Incidence of early AMD (n = 248)	Incidence of late AMD (n = 76)
0 (n = 562)	1.0 (reference)	1.0 (reference)
*n* (*%*)	*72* (*14.6*)	*18* (*3.2*)
1 (n = 860)	1.05 (0.76–1.44)	1.29 (0.72–2.34)
*n* (*%*)	*111* (*15.2*)	*35* (*4.1*)
2 (n = 372)	1.39 (0.94–2.06)	1.42 (0.70–2.87)
*n* (*%*)	*53* (*16.8*)	*17* (*4.6*)
3 (n = 89)	1.46 (0.69–3.08)	2.11 (0.67–6.67)
*n* (*%*)	*9* (*12.3*)	*5* (*5.6*)
4 (n = 20)	1.51 (0.33–6.81)	4.25 (0.49–36.73)
*n* (*%*)	*3* (*15.8*)	*1* (*5.0*)
*P-value* for linear trend	0.08	0.11

^a^Adjusted for age, sex, white cell count, and fish consumption.

## Discussion

We provide novel epidemiological evidence showing that the collective effect of poor health behaviours (smoking, low fruit and/or vegetable consumption, high intake of alcohol and low levels of physical activity) on AMD was substantial in our older cohort. At baseline, BMES participants who engaged in all 4 poor health behaviours had a 5- and 29.5-fold greater odds of any and late AMD, respectively, compared to those exhibiting none of these behaviours. When examining the lifestyle factors separately, only 3 of the 4 poor health behaviours were associated with the prevalence and/or incidence of AMD, that is, physical activity by itself was not independently associated with AMD.

Lifestyle factors such as smoking, dietary habits, physical activity, and alcohol consumption were independently associated with AMD risk in numerous studies^2, 5, 8–12, 14^, but few studies have investigated the combined impact of these factors. Data from both the BMES and CAREDS^2, 9^ suggest that broadly a combination of lifestyle practices might have a more important effect on the likelihood of AMD than focusing on certain isolated components of an individual’s lifestyle. We hypothesise that adopting a number of poor health behaviours could contribute to increasing oxidative stress, inflammation, and worsening blood lipid levels, all of which are thought to be pathogenic mechanisms that promote AMD^9^. Specifically, unhealthy lifestyles could increase AMD risk by promoting systemic inflammation, which is widely thought to contribute to AMD pathogenesis. Poor dietary patterns and physical activity have been related to higher blood levels of C-reactive protein, a marker of systemic inflammation^8, 9, 14, 28^. Systemic risk factors such as obesity, cardiovascular risk factors (e.g. hypertension) and cardiovascular disease have been linked with AMD^10^, and there is robust evidence to show that collective health behaviours have a strong influence on the risk of these conditions^19, 29^. Therefore, this is another potential pathway by which engaging in a number of poor health behaviours could modify the likelihood of developing or worsening of existing AMD.

In addition to these mechanisms, we hypothesize that smoking, inadequate physical activity and low intakes of fruits and vegetables might collectively increase the prevalence of AMD by diminishing the status of macular pigment. Macular pigment density was shown to be associated with a combination of health behaviours in CAREDS^9^. The carotenoids composing macular pigment can block the frequencies of blue light that are known to damage the retina directly; they may also quench reactive oxygen species that form as a result of the light- and oxygen-rich environment^9, 30^. Lutein and zeaxanthin supplementation from foods can clearly increase macular pigment density^31, 32^. Several aspects of diet such as the overall intake of fruits, vegetables, whole grains, and fat may contribute to the uptake and turnover of these carotenoids^9, 33^. Physical activity might also contribute to greater macular pigment density by reducing inflammation and oxidative stress directly or by reducing obesity^9^. Obesity is related to lower macular pigment density^33^ and may increase oxidative stress and carotenoid turnover as well^9, 34^. Finally, smoking has been shown to reduce macular pigment optical density, which reflects levels of the protective carotenoids in the macula^35^.

While the odds of any, early and late AMD increased significantly as the number of poor health behaviours increased, we caution that the confidence intervals were large and thus, less precise, reflecting the very small numbers in some groups i.e. particularly the group of participants who engaged in all 4 poor health behaviours. Moreover, the small sample size (and possibly reduced statistical power) could explain the largely non-significant findings related to 15-year incidence of AMD. Therefore, larger studies or pooled samples across many studies are warranted to provide additional evidence and more reliable risk estimates for the associations between combined lifestyle behaviours and the prevalence and incidence of AMD.

Nevertheless, our study findings appear to align with current advice regarding a number of other chronic diseases (e.g. cardiovascular disease and diabetes), specifically, quitting smoking, exercising more, eating plenty of vegetables and fruits, and moderating the intake of alcohol^5, 9^. Thus, BMES data provide further supportive evidence indicating that even modest differences in lifestyle, could make a significant difference to health at both the individual and population level. Consequently, this study underscores the importance of proper patient education on recommendations of a package of healthy lifestyle behaviours and regular follow-up by the appropriate clinicians to ensure that these recommendations are being implemented routinely and correctly^36^. Moreover, lifestyle-focused healthcare could then be achieved through appropriate co-management between eye-care practitioners and other health professionals, such as dieticians, to achieve desirable lifestyle changes^37^.

Strengths of this study include its prospective design; long-term follow-up of a stable population-based sample; and collection of robust data on major confounders and lifestyle parameters. Further, this study uses high quality stereoscopic retinal photography with validated grading to assess macular conditions^26, 38^. However, there are some noteworthy limitations. First, there is potential for survival bias in our study as persons with AMD who may have quit smoking or improved their diet could have been lost to follow-up or have died; as such, observed associations need to be interpreted with caution. Second, because we have examined several associations, the possibility of chance findings cannot be discounted. Finally, we cannot disregard the possibility of residual confounding from factors that were not measured or known, which could have influenced lifestyle practices and/or AMD risk of study participants.

In summary, this cohort study provides unique data showing that a combination of unhealthy behaviours including: smoking, poor diet, high alcohol intake and low physical activity, was associated with a markedly higher likelihood of AMD. Our findings, however, require confirmation in intervention studies and in larger, long-term population-based studies that include a broader sample of ethnic backgrounds. For the time being, motivating patients with AMD to eat better, exercise more, limit alcohol intake and avoid smoking seems advisable to decelerate the development or worsening of existing AMD.
